# Assessing streetscape greenery with deep neural network using Google Street View

**DOI:** 10.1270/jsbbs.21073

**Published:** 2022-02-25

**Authors:** Taishin Kameoka, Atsuhiko Uchida, Yu Sasaki, Takeshi Ise

**Affiliations:** 1 Center for the Promotion of Interdisciplinary Education and Research, Kyoto University, Kitashirakawaoiwake-cho, Sakyo-ku, Kyoto 606-8502, Japan; 2 Field Science Education and Research Center, Kyoto University, Kitashirakawaoiwake-cho, Sakyo-ku, Kyoto 606-8502, Japan

**Keywords:** urban greenery, green view index, Google Street View, deep learning, chopped picture method, GIS

## Abstract

The importance of greenery in urban areas has traditionally been discussed from ecological and esthetic perspectives, as well as in public health and social science fields. The recent advancements in empirical studies were enabled by the combination of ‘big data’ of streetscapes and automated image recognition. However, the existing methods of automated image recognition for urban greenery have problems such as the confusion of green artificial objects and the excessive cost of model training. To ameliorate the drawbacks of existing methods, this study proposes to apply a patch-based semantic segmentation method for determining the green view index of certain urban areas by using Google Street View imagery and the ‘chopped picture method’. We expect that our method will contribute to expanding the scope of studies on urban greenery in various fields.

## Introduction

The importance of greenery in urban areas has been discussed from both ecological and esthetic perspectives. For instance, Sharifi (2020) reviewed 56 academic articles published until October 2019 to explore the co-benefits and synergies between adaptation and mitigation strategies for the effect of climate change in urban areas and emphasized the key role of green infrastructure in the conclusion. Greenery could make urban areas comfortable not only by mitigating heat but also by contributing to preferable streetscapes (Klemm *et al.* 2015). In recent years, public health and social scientific perspectives have demonstrated the benefits of urban greenery for people’s lives. For example, greenery on the street could encourage local people to walk around the neighborhood (Ki and Lee 2021, Lu *et al.* 2018), which could improve their health and prevent obesity (Li and Ghosh 2018). From a social scientific perspective, greenery on the street could have a positive influence on interactions among local people (Hong *et al.* 2018, Wang and Vermeulen 2020). These recent empirical studies on urban greenery were enabled by a combination of ‘big data’ of streetscapes and automated image recognition.

Since the beginning of the service in the US in 2007, Google Street View (GSV; Google LLC 2021) has been widely used to observe streetscapes around the world. The GSV application offers interactive 360-degree panorama views from the street in combination with geographical data. The images used in GSV are usually taken in daylight by using a camera mounted on top of cars, at a height of approximately two meters from the ground. The panorama views seem to have intervals of several meters. However, the imagery data tends to be intense in urban areas, and the update frequency of the imagery seems to be uneven, even within the same region.

Since mid-2010, automated image recognition has been applied for the identification of objects in GSV images. Li *et al.* (2015) developed a method for estimating the green view index (GVI) of target urban areas more easily by using GSV instead of conventional methods. The GVI is an evaluation metric of visible greenery from the perspective of pedestrians on the street (Yang *et al.* 2009). They collected GSV images of the target urban areas and extracted regions with green vegetation by classifying green areas on the images. Subsequently, the GVI of the target area was reported as the average proportion of green vegetation. However, they mentioned that this image recognition method could also detect green objects other than vegetation, such as road signs or advertisements, although their proportion in the streetscapes seemed to be too small to influence the results. Semantic segmentation as a machine learning method is another effective tool that classifies areas of certain objects as well as colors in an image. This method has been applied to identify greenery and other objects in GSV images in urban regions (e.g., Ki and Lee 2021, Wang and Vermeulen 2020, Ye *et al.* 2019). Object detection models are also applied for high-resolution GSV images to detect individual instances of plants of certain species along roadways (Ringland *et al.* 2021). However, for such methods, significant efforts are usually required to train machine learning models, especially in the process of annotation. To ease the burden on humans for this process, generating patches from annotated images for training models can be an effective approach, as Ise *et al.* (2018) and Ringland *et al.* (2019) performed it to classify multiple species of mosses and crops, respectively.

In the present study, we propose the application of the method of determining the GVI of certain urban areas using GSV imagery and a deep learning method to ameliorate the weaknesses of existing methods Guo *et al.* (2018) and Li *et al.* (2015) mentioned. This could contribute to expanding the scope of studies on urban greenery in various fields.

## Materials and Methods

### Collecting streetscapes on Google Street View

To obtain the imagery of streetscapes in the target districts of an urban area, we downloaded GSV image data via Google’s application programming interface (API). The API provides GSV images by referring to several parameters, including the coordinates of the location, vertical and horizontal angles, and the size of the image. We extracted the coordinates of places where people would be living using open data provided by the government.

**Fig. 1.**
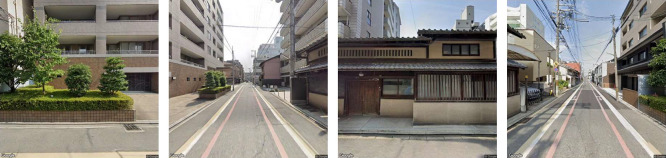
A set of Google Street View images from a point in a target district. The angles of the four images were rotated by 90°.

First, we referred to the census data (Statistics Bureau, Ministry of Internal Affairs and Communications of Japan 2021) of Kyoto city, Kyoto prefecture, Japan, and randomly extracted five percent of the districts defined as *Densely Inhabited District* in the census by ward. In this way, 224 districts were selected. Second, we combined the geographical boundaries in the census and polygon data of buildings in the target districts (Geospatial Information Authority of Japan 2016) by using QGIS 3.16.3 (QGIS Development Team 2021) and listed the center of gravity of the buildings in the district. To avoid collecting images only around roadways connecting residential areas, we used buildings such as houses as reference coordinates in the following process. Third, GSV images of the 224 target districts were collected from March 25 to 26, 2021 by referring to the list of coordinates. Regarding the other parameters for the API, the image size was set as 640 × 640 pixels, which is the largest size allowed in the free plan of the API. The location was limited to outdoors, and the vertical angle of the images was set as horizontal. We tried to extract four images from each coordinate, that is, at angles of 0°, 90°, 180°, and 270° to obtain a nearly panoramic view from a certain point on the GSV (Fig. 1 shows an example).

Finally, blank or duplicate images were excluded from the collected images. Although the API of GSV offered the latest version of the imagery for each coordinate at that moment, gaps of several years could occur between the shooting dates of these coordinates, sometimes even within the same district. Because the API for collecting images did not supply metadata with imagery, including the shooting date, the correct gaps could not be calculated from the image data collection. Additionally, because the API offered images taken at the nearest point from the coordinate input, the same images could be downloaded from different coordinates when they had the same nearest point where the images existed. Blank images could also be obtained from points whose nearest point of imagery was considerably far away. Consequently, 47,104 images were collected, and the number of collected images differed across districts, ranging from 4 to 1,380 (M = 210, SD = 209).

### Semantic segmentation and calculation of green view index

Convolutional neural networks (CNNs) are widely used in various visual recognition problems (Noh *et al.* 2015), this method is applied to image classification (Sharma *et al.* 2018) and semantic segmentation (Kampffmeyer *et al.* 2016). Image classification using deep learning assumes that a consistent contour structure exists for target objects. However, the greenery in the GSV images often exhibits an amorphous shape. Semantic segmentation has been applied for tasks concerning such amorphous objects such as cracks on the structure (Dong *et al.* 2020) and cancer subtypes (Hou *et al.* 2016). Semantic segmentation, therefore, can be the most appropriate method for building an AI model for estimating the GVI.

**Fig. 2.**
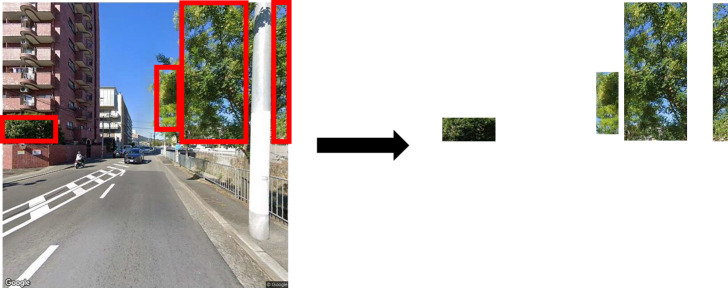
Example of the trimming process for greenery by a human rater.

**Fig. 3.**
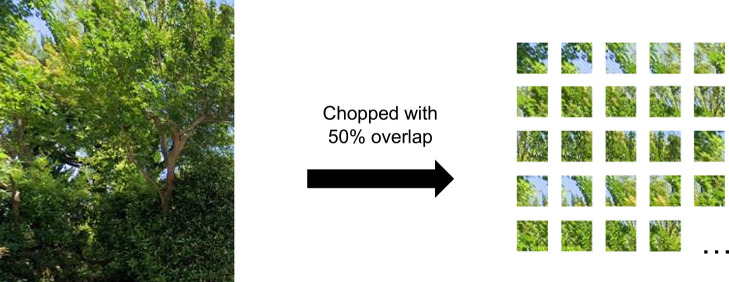
Greenery image chopped into 32 × 32 pixels squares.

Therefore, we employed the chopped picture method (CPM: Ise *et al.* 2018). This is a type of method by a patch-based semantic segmentation using a CNN such as Hou *et al.* (2016) with model training. The method is efficient for identifying amorphous objects such as vegetation (Watanabe *et al.* 2020). Furthermore, it is remarkable that CPM requires less effort to generate numerous training datasets from fewer images. Recently, various techniques have been tested to implement model training with less effort. To alleviate the need for large-scale detailed annotations, weakly supervised semantic segmentation techniques have been developed (Bearman *et al.* 2016), for example, image-level labels (Ahn and Kwak 2018), points (Bearman *et al.* 2016), and bounding boxes (Khoreva *et al.* 2017, Papandreou *et al.* 2015). Additionally, CPM is a type of weakly supervised method in this research, our method makes one or several rectangles for the target object in images (Fig. 2) and labels them as greenery or no greenery (e.g., building, car, sky). Although a rectangle is easier for training dataset preparation than standard semantic segmentation, the method (Fig. 2) is not dramatically different from the other weakly supervised methods in reducing time and labor. However, CPM can generate many smaller square patches as training images (Fig. 3) from a rectangle (Fig. 2) which dramatically saves time for training dataset creation. This advantage indicates creating and revising AI models is comparatively easy. This study, therefore, selected CPM to develop an AI model for estimating GVI.

The details of the target object that the models could identify depend on the resolution of the target images. Considering the low resolution of GSV imagery (640 × 640 pixels), the present study focused only on greenery and ignored differences in vegetation types and conditions.

The greenery coverage rate of each collected image was calculated using a machine-learning model. For model training, one or two target districts were randomly extracted from each ward, depending on the total number of target districts within each ward, and 3,528 images were separated as training datasets from the collected images. Using the training dataset, we built a machine learning model to identify greenery in the remaining collected images used as the target dataset.

At the beginning of the training process, areas of living leaves (greenery) in the images of the training dataset were trimmed for positive imagery such as street trees and garden trees (Supplemental Fig. 1), and areas of the other streetscape elements such as houses, utility poles, sky and roads were clipped as negative imagery (Supplemental Fig. 2). In this process, human raters trimmed the target objects as a rectangle in a GSV image of the training dataset. For the greenery, one or several rectangles were trimmed from a single image (Fig. 2). The positive dataset comprised 115 rectangles, and the negative dataset contained 743 rectangles. Subsequently, the rectangles were automatically chopped into smaller square patches (32 × 32 pixels) with 50% overlap, both vertically and horizontally (Fig. 3 shows an example), using the programming language R 3.4.4 (R Core Team 2018). Thus, 86_,_785 smaller square patches were created for training the model.

**Fig. 4.**
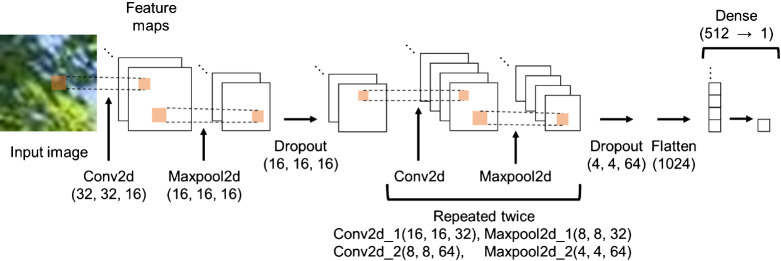
Schematic of the convolutional neural network architecture.

Finally, the model was trained with the smaller square patches using the deep learning framework Keras on Python 3.6.9, running on Ubuntu 18.04. The CNN was applied, and its architecture is shown in Fig. 4.

**Fig. 5.**
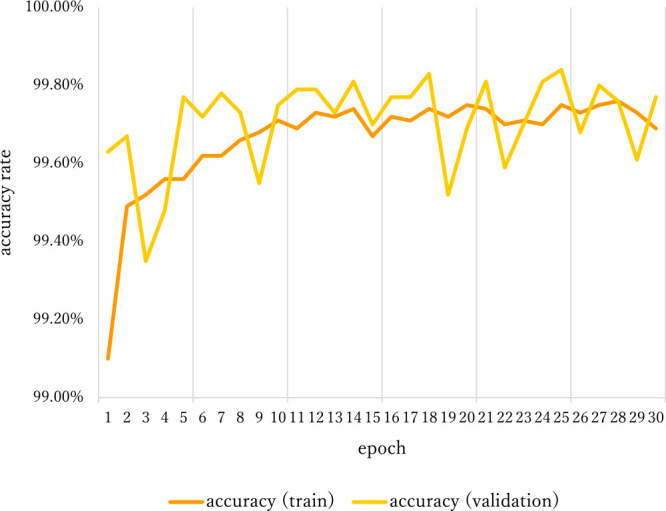
Accuracy output of training for object identification of greenery.

**Fig. 6.**
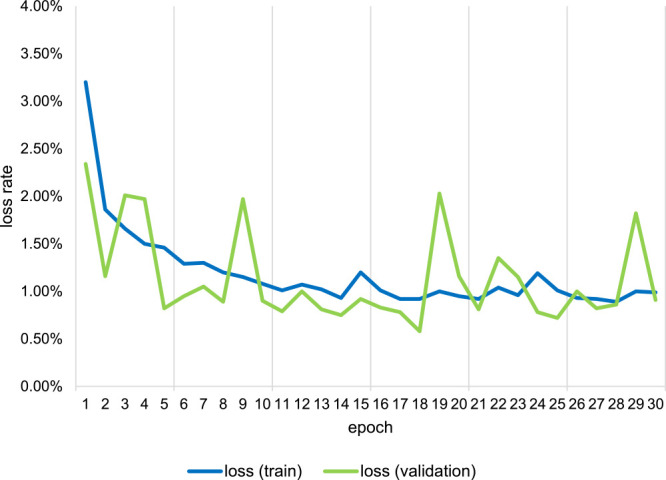
Loss output of training for object identification of greenery.

The batch size was set to 32. The optimizer used was Adam (Kingma and Ba 2015), and the learning rate was set at the default. The computer used in this study had an Intel Core i7-8700K processor, an NVIDIA GeForce RTX2060 GPU, and 32 GB RAM. For validation, we used 20% of the smaller square patches. The model was trained for 30 epochs, and its training accuracy reached 99.7% (Figs. 5, 6). Since the difference in appearance between greenery and the others (Supplemental Figs. 1, 2) is obvious, the training accuracy of the model was high enough in epoch 1 (Fig. 5). Additional epochs didn’t make any substantive changes.

**Fig. 7.**
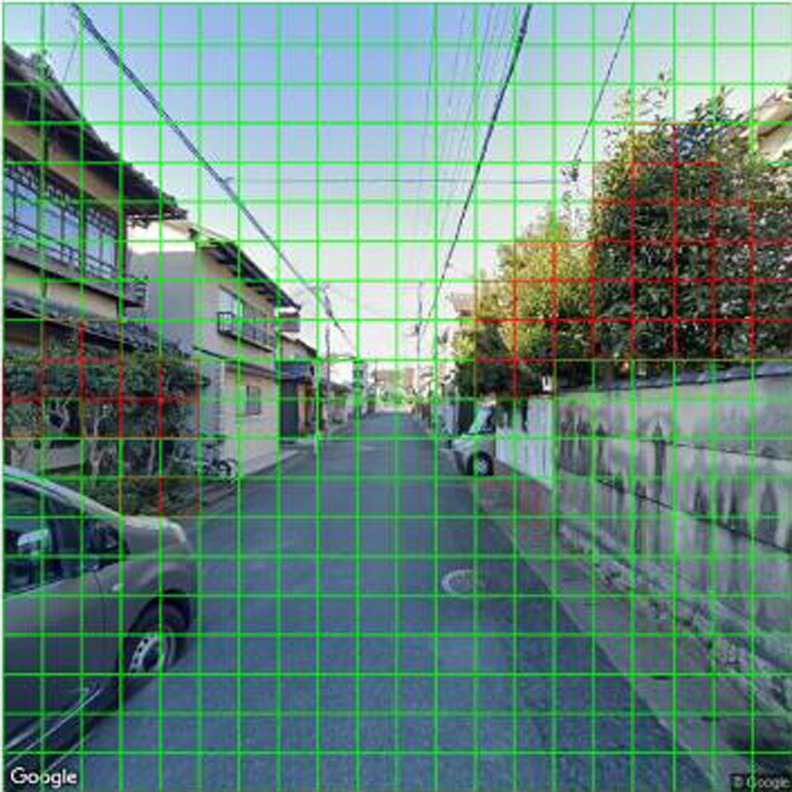
Streetscape image processed by the deep learning model. Red square cells were the objects identified as greenery by the model (16.25%), and green square cells were the other objects.

Using the built model, we recognized the areas of living leaves in each image of the target dataset. As shown in Fig. 7, the model outputs a binary outcome (i.e., positive or negative for the target object) of recognition of each square cell composed of an image of the target dataset and the percentage of positive square cells in an image. Subsequently, we averaged the positive rate of the images by using the district of the target dataset as its GVI.

### Process of Evaluation

**Fig. 8.**
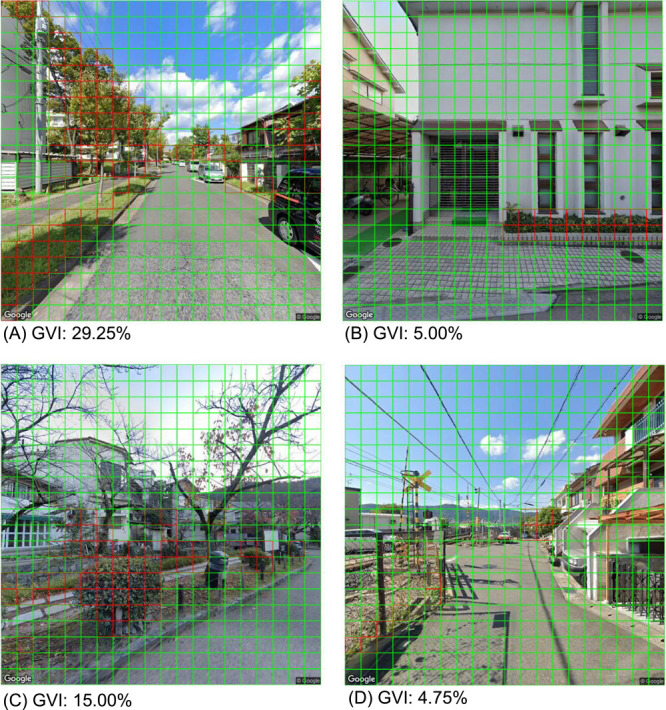
Streetscape images processed by the deep learning model. (A) effect of shadow (%), (B) effect of artificial green (%), (C) effect of leafless trees and autumn leaf (%), (D) effect of distant mountain (%).

For the evaluation, we calculated the overall accuracy, precision, and recall. Four raw GSV images including 1,600 square cells in total that were not used in the training were extracted (see Fig. 8), and a 20 by 20 grid with identical square cells (32 × 32 pixels) highlighting the output acquired by the model was placed on each image. A human rater who did not participate in creating positive greenery rectangles for training, independently evaluated the square cells of the images as positive, if the greenery seemed to occupy more than a half of the cell, and subsequently, the remaining cells were labeled as negative. Eventually, we compared the results determined by the human rater with those determined by the model, and confirmed the correspondence between the position of every pair of the cells in the GSV image.

### Color-based classification of greenery for comparison

To demonstrate the advantage of CPM in the classification performance of greenery on GSV imagery over a previous method, we also applied an automatically unsupervised classification of greenery for the raw GSV images used in the evaluation process. In this process, we reproduced the color-based method developed by Li *et al.* (2015) which introduced a means of estimating GVI by using GSV imagery in the early years. This method focused on the spectral information of greenery to classify, that is, high reflectance at the green band and relatively low at red and blue bands in the Red-Green-Blue color model. We followed the workflow shown by them to detect the differences of those color bands, using R 3.4.4 (R Core Team 2018) as the programming language. The threshold of the difference, an undefined parameter in their article, was set to 0.05 by trial and error. Eventually, areas of greenery in the target GSV images were defined based on that threshold.

## Results

### The coverage ratio of greenery in Kyoto city using the machine learning model

By using this model, we were able to extract the green parts of street trees and garden trees from the GSV. Fig. 8 shows an example of the output by the model. The results of the evaluation, wherein we compared the results of a human rater and the model, were acceptable. The overall accuracy was 0.96; the precision was 0.73; and the recall was 0.92.

Regarding the details of the output by the model, Fig. 8A depicts the successful extraction of greenery from the shadows of a tree. The effects of artificial green objects, such as artificial turfs (Fig. 8B), were suppressed. Moreover, leafless trees, autumn leaves (Fig. 8C), and distant mountains (Fig. 8C, 8D) were smoothly excluded, although we were concerned that they might be identified as false positives.

Meanwhile, if greenery occupied only a small proportion of the square cell, or dark leaves or grass occupied, different judgement between our AI model and the human rater tended to occur (Fig. 8A, 8C, Supplemental Figs. 3–6).

The GVI in Fig. 8A, 8B, 8C, and 8D were 29.25%, 5.00%, 15.00%, and 4.75%, respectively.

### Comparison between CPM and color-based classification

**Fig. 9.**
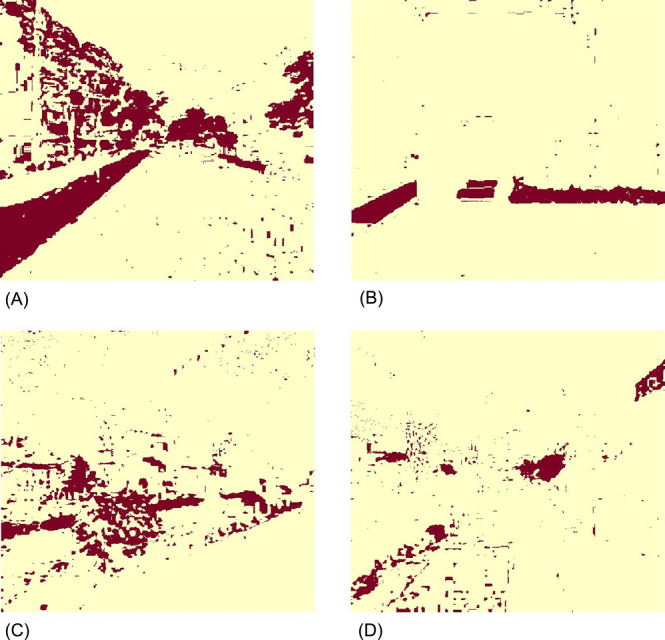
Greenery extraction results by a color-based method of Li *et al.* (2015). The brown areas showed greenery based on the differences of color bands in the Red-Green-Blue color model.

The results of greenery extraction by a color-based method of Li *et al.* (2015) were shown in Fig. 9. Compared with the corresponding images in Fig. 8, a more detailed contour of the greenery in those images was extracted as brown areas in Fig. 9. However, green artificial objects and shadows were wrongly extracted (e.g., green mats in Fig. 9C and shadows of the fence in the forefront of Fig. 9D), which was a limitation of this method as mentioned by Li *et al.* (2015). Moreover, this model also classified some shadows as greenery, though tiny noises of those were supposed to be filtered in their method.

### Features of GSV

**Fig. 10.**
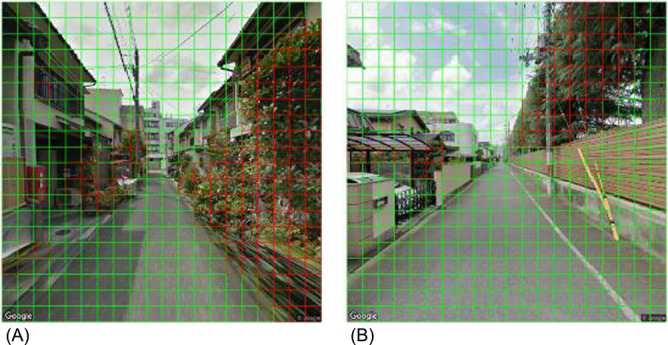
Streetscape images processed by the deep learning model. (A) an error caused by blurred images, (B) error caused by low resolution.

Generally, calculating GVI requires a large-scale field survey. However, by using GSV, we were able to acquire 47,104 images over 224 areas in Kyoto City. The GSV survey covered a wide range of survey areas and allowed us to easily acquire data. However, as shown in the lower right and lower left of Fig. 10A, blurred images were sometimes incorrectly classified. In addition, because of the low resolution of the images, it was not possible to detect greenery at the back of the screen, as shown in Fig. 10B.

### Mapping of GVI of the target districts in Kyoto city

**Fig. 11.**
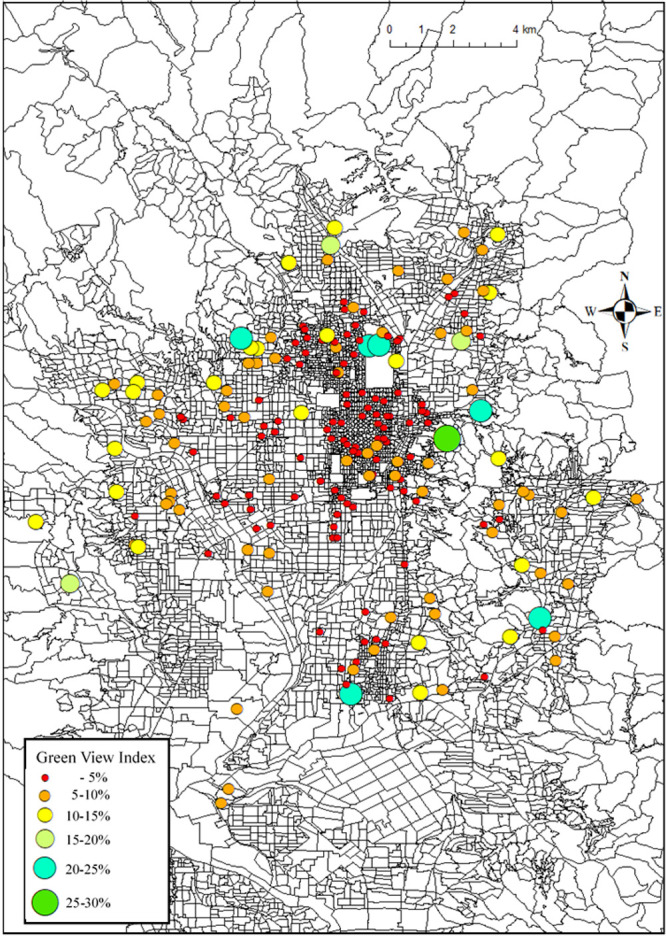
Mapping of GVI of the target districts in Kyoto city.

Finally, we mapped the GVI of the 196 districts where we were able to obtain at least 10 images per district from the target dataset using ArcGIS 10.8.1 (Fig. 11). In the map, the GVI of the target districts tended to increase from the central area to the suburbs. In the Nakagyo Ward of Kyoto City, the GVI was predominantly less than 10%, while even in the city center, there were places where the GVI exceeded 20% owing to the park and its hedges. By mapping the GVI, we were able to examine the overall trend and specific points of the GVI in Kyoto City.

## Discussion

The purpose of this study was to propose a simpler method for calculating GVI in cities than existing methods. In existing methods, semantic segmentation is the primary approach for calculating GVI. However, because most semantic segmentation methods are dependent on numerous images with detailed semantic segmentation masks, it is considerably labor intensive to manually annotate these masks. Such annotations are time-consuming, frustrating, and commercially expensive (Guo *et al.* 2018).

In this study, we suggest using CPM (Ise *et al.* 2018, Watanabe *et al.* 2020) as an alternative to standard semantic segmentation. It is suitable for objects with amorphous shapes. The time and labor required to create training images for CPM are significantly reduced compared to standard semantic segmentation. The CPM is a type of patch-based semantic segmentation. In this method, to create training images, we selected rectangular areas that include the target object. The rectangles are divided into small square patches (i.e., 32-pixel squares). Among these patches, we manually selected only the patches with >50% area covered by the target object. By using this method, we automatically created hundreds of training image candidates, and quickly screened and removed inappropriate images (>50% of area covered by other objects). Ordinary methods of semantic segmentation tend to require significant time and effort for annotation (Bearman *et al.* 2016). In contrast, we were able to produce 86,785 training images (small square patches) from 858 rectangles that contained the target object. The users of this method are only required to make those rectangles. This approach significantly reduces the time and labor required to generate the training images, and the loss of model performance compared to standard segmentation is minimal. Furthermore, this method can be implemented with effective CNNs such as LeNet (Lecun *et al.* 1998) and AlexNet (Krizhevsky *et al.* 2012); moreover, the computational efficiency is higher than that of standard semantic segmentation.

The CPM has been used for moss classification using digital cameras (Ise *et al.* 2018) and bamboo detection using Google Earth (Watanabe *et al.* 2020). However, we found that GSV was also effective for detecting greenery in urban areas. By combining the CPM with GSV images, we were able to show that it is possible to create a machine learning model that can more easily detect greenery and calculate GVI than existing methods. Thus, we were able to propose an easier method for calculating GVI in urban areas than existing methods.

### Output of evaluation

The overall accuracy, precision and recall were 0.96, 0.73 and 0.90, respectively in our AI model. The result of the overall accuracy shows that our AI model has adequate accuracy using GVI investigation. Additionally, the result of the recall shows few false negatives. However, the precision was relatively lower than the overall accuracy, and the recall shows our AI model has false positives to a certain degree. The primary reason for the moderate precision compared with the recall, is that the model seemed to be more sensitive to sparse or dark leaves or grass than the human rater (Supplemental Figs. 3–6 show the details). Specifically, our AI model regarded the square cells that occupied less than 50% by greenery as positive, although the human rater regarded those cells as negative. This result indicates that the human rater and our AI model possess different judgement levels if square cells have greenery but were not fully occupied by greenery such as tree heads and branch tips. This situation would be improved to remake the criteria of our AI model related to greenery occupancy. For example, our current AI model tends to regard the square cells which were not more than 50% occupied by greenery as positive, but it is possible to remake our AI model into a new AI model which regards the cells that occupied over 50% by greenery as greenery. As a result, the precision of the new model is expected to be improved, our model can more accurately calculate GVI.

### Strength and weakness of the machine learning model combining the CPM and GSV images

In a previous study using color-based classification (Li *et al.* 2015), it was observed that artificial green and shadows were falsely detected as greenery. We tested the same images using both CPM and the method of greenery extraction by a color-based method of Li *et al.* (2015); the results (Fig. 9C, 9D) showed the same weak points as mentioned by Li *et al.* (2015). Comparing those images (Fig. 9) with the corresponding images in Fig. 8, these tendencies were suppressed in CPM. This is because our method can implement a machine learning model that includes identification by using information about the texture of the object as well as color. Because artificial green regions and shadows are common in urban areas, CPM can be expected to be one of the most useful methods for conducting GVI surveys in urban areas.

In recent years, GVI calculations have been used in various scenarios, such as research and government reports. In the future, the standards for calculating GVI may change depending on the purpose and features of the survey areas. For example, it may be necessary to detect mountains and weeds in a distant view as greenery. The annotation process of semantic segmentation is quite time-consuming, and minor changes will take time. Therefore, because our method can efficiently produce training images, it is easy to expand the detection target. This is a useful method for model creation and extension.

Herein, we demonstrated the feasibility of the CPM and GSV for the assessment of greenery in Kyoto city. We showed that our method can provide helpful information on greenery in urban areas, which was not previously understood. However, several issues remain to be resolved.

The first issue is false positives for square cells with only a small proportion of greenery, such as tree heads and branch tips or those cells occupied by dark leaves or grass (Figs. 8, 10B, Supplemental Figs. 3–6). It could be solved to remake our AI model into a new AI model which has similar criteria of a human rater or to analyze them more precisely by using high-resolution images. The second issue is that the GSV image contained blurred images (Fig. 10A). At present, we have to manually remove these images. In the future, we would like to develop a method to automatically remove them.

In this study, we focused on the CPM and used GSV to suggest the application of the method of determining the GVI of certain urban areas by using GSV imagery and a deep learning method to ameliorate the weaknesses of existing methods. We expect that our method can form the foundation for future social activities and academic research on GVI research improvement.

## Author Contribution Statement

TK collected and analyzed data using GIS software and wrote the Results and Discussion sections. AU designed the study, collected and analyzed the data by using the API and the machine learning model, and wrote the Introduction and Materials and Methods sections. YS trained a machine-learning model. TI guided all the steps of the experiments and manuscript preparation.

## Supplementary Material

Supplemental Figures
