# Supplementary material for: Assessing streetscape greenery with deep neural network using Google Street View
Source: Breed Sci. 2022 Feb 25;72(1):107–14. doi: 10.1270/jsbbs.21073 (PMC8987839; doi:10.1270/jsbbs.21073)
Supplement: Supplementary file 1 — Supplemental Figures [file 72_107_s1.pdf]

Kameoka et al./Breeding Science
doi: 10.1270/jsbbs.21073

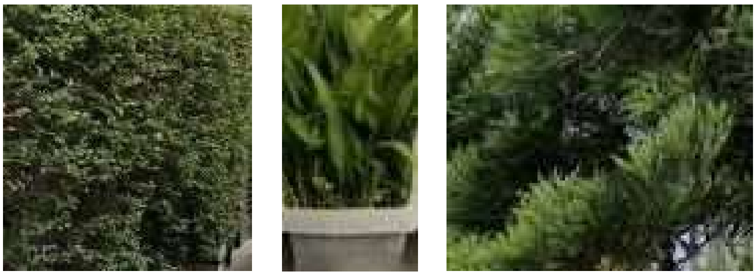

Supplemental Fig. 1. Trimmed positive imagery as training dataset. The positive and negative dataset as training dataset Supplemental Figure 1 to 2 show some positive and negative training dataset. Areas of living leaves (greenery) such as street trees and garden trees in the images of the training dataset were trimmed for positive imagery, and areas of the other streetscape elements such as houses, utility poles, sky and roads were clipped as negative imagery.

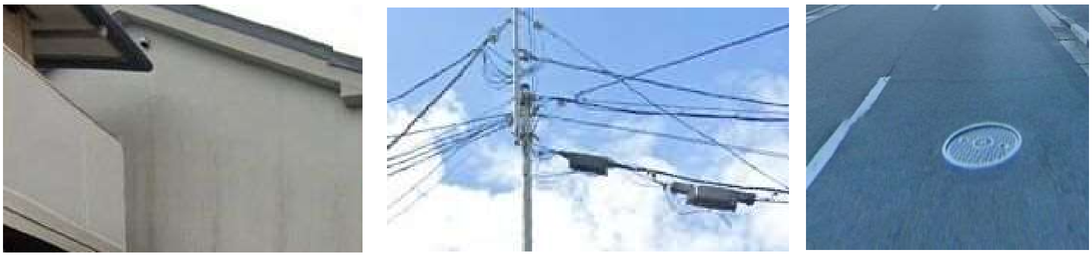

Supplemental Fig. 2. Trimmed negative imagery as training dataset.

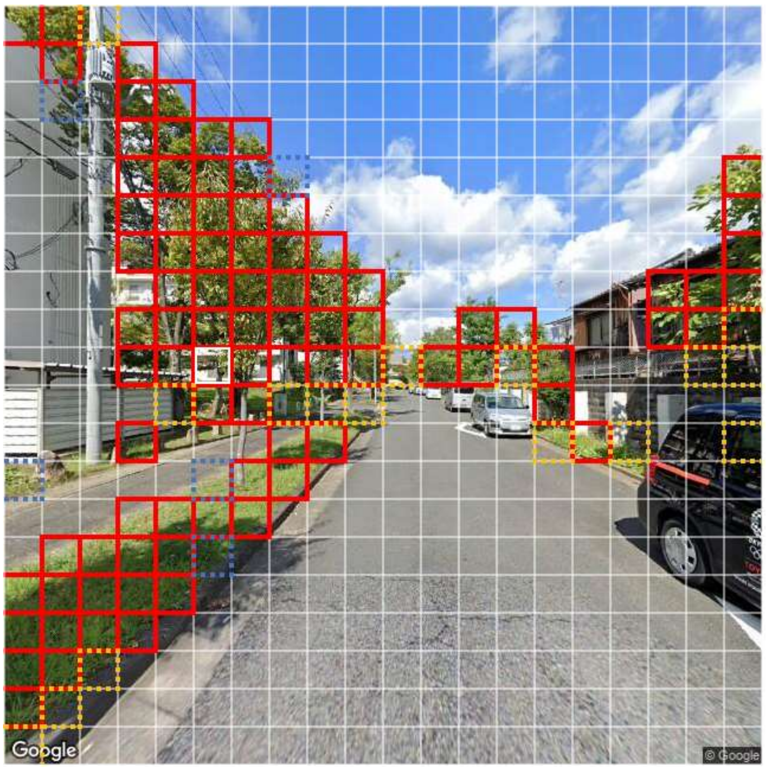

Supplemental Fig. 3. The human rater's judgement and false classification on Figure 8 (A). The human rater's judgement on four GSV images of Figure 8. Supplemental Figure 3 to 6 show comparisons between the human rater's judgement and the model's classification for greenery. Red square cells mean true positive, while white square cells mean true negative. Orange dot lined square cells mean false positive, while blue dot lined square cells false negative. These figures show that false classification conducted by the model tended to happen for positive rather than negative.

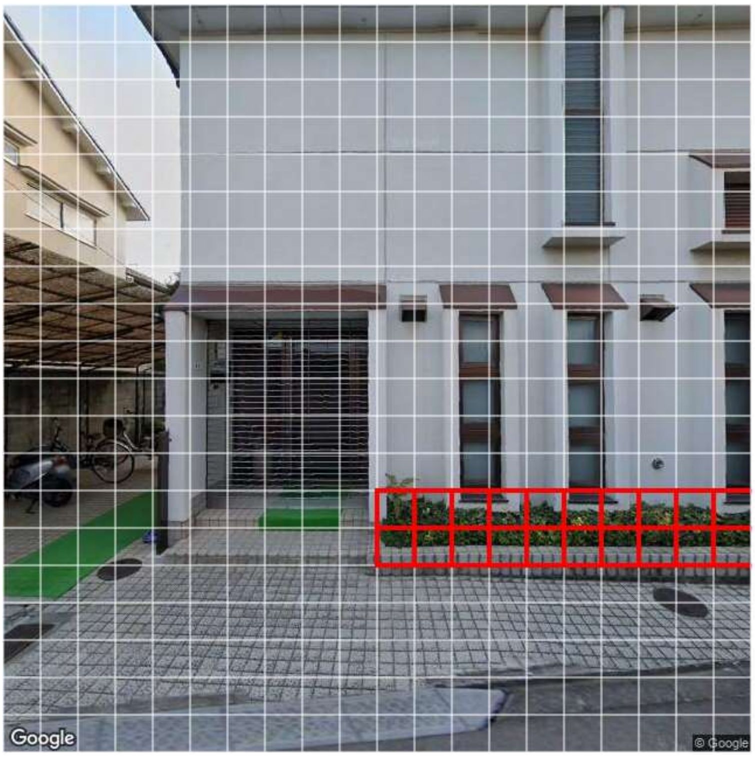

Supplemental Fig. 4. The human rater’s judgement and false classification on Figure 8 (B).

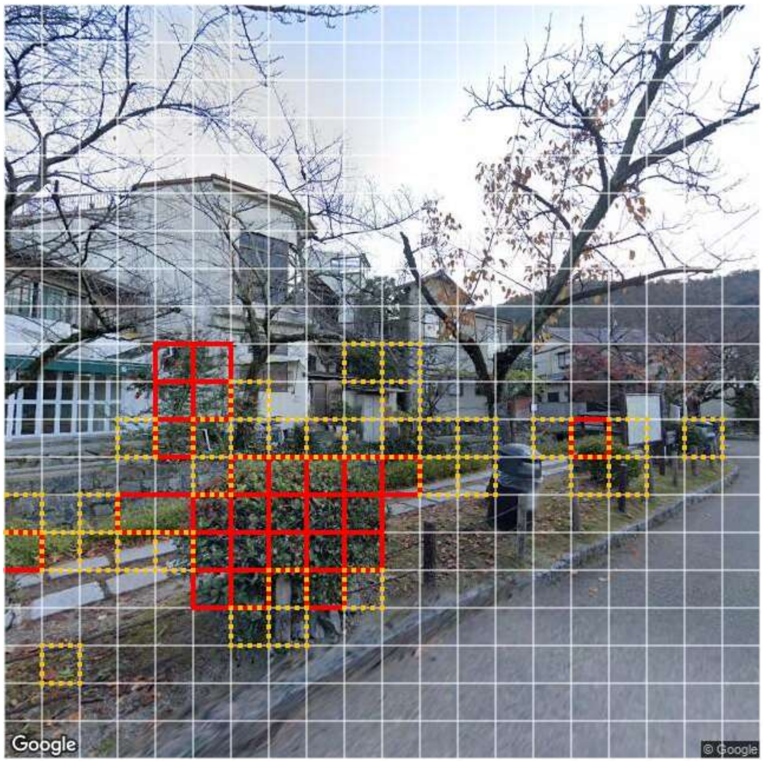

Supplemental Fig. 5. The human rater’s judgement and false classification on Figure 8 (C).

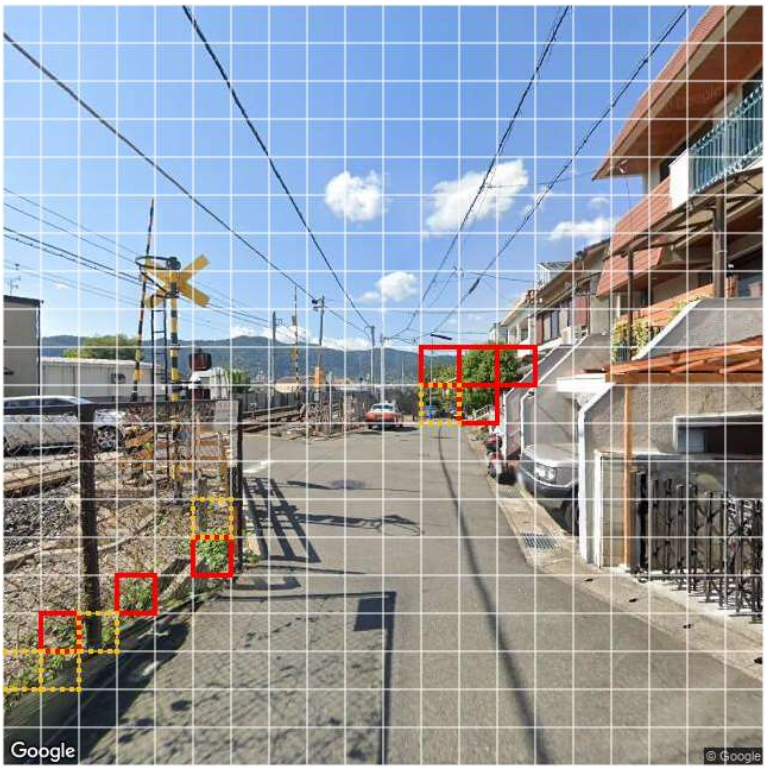

Supplemental Fig. 6. The human rater's judgement and false classification on Figure 8 (D).
